# Efficacy of Four *Solanum* spp. Extracts in an Animal Model of Cutaneous Leishmaniasis

**DOI:** 10.3390/medicines5020049

**Published:** 2018-06-05

**Authors:** Paul Cos, Jo Janssens, Abel Piñón, Osmany Cuesta-Rubio, Arianna Yglesias-Rivera, Alexis Díaz-García, Wagner Vilegas, Lianet Monzote

**Affiliations:** 1 Laboratory of Microbiology, Parasitology and Hygiene (LMPH), Faculty of Pharmaceutical, Biomedical and Veterinary Sciences, Antwerp University, 2000 Antwerpen, Belgium; paul.cos@uantwerpen.be (P.C.); jokejanssens5@hotmail.com (J.J.); 2 Parasitology Department, Institute of Tropical Medicine “Pedro Kouri”, Havana 10400, Cuba; thelma@ipk.sld.cu; 3 Academic Unit of Chemical Sciences and Health, Technical University of Machala, Machala 070101, Ecuador; osmanycuesta@yahoo.com; 4 Research Department, Laboratories of Biopharmaceuticals and Chemistries Productions (LABIOFAM), Havana 10400, Cuba; ariannay@ipk.sld.cu (A.Y.-R.); alediaz@ipk.sld.cu (A.D.-G.); 5 UNESP—São Paulo State University, Coastal Campus of São Vicente, São Paulo 19014-020, SP, Brazil; wagner.vilegas@unesp.br

**Keywords:** *Leishmania amazonensis*, cutaneous leishmaniasis, BALB/c mice, *Solanum havanense*, *Solanum myriacanthum*, *Solanum nudum*, *Solanum seaforthianum*

## Abstract

**Background:** Leishmaniasis is a complex protozoa disease caused by *Leishmania* genus (Trypanosomatidae family). Currently, there have been renewed interests worldwide in plants as pharmaceutical agents. In this study, the *in vivo* efficacy of *Solanum* spp. is assessed in an *L. amazonensis* BALB/c mice model for experimental cutaneous leishmaniasis. **Methods:** Animals were infected with 5 × 10^6^ metacyclic promastigotes and 30-day post-infection, a treatment with 30 mg/kg of *Solanum* extracts or Glucantime^®^ (GTM) was applied intralesionally every four days to complete 5 doses. **Results:** Neither death nor loss of weight higher than 10% was observed. All the tested extracts were able to control the infection, compared with the infected and untreated group. *Solanum havanense* Jacq. extract showed the highest efficacy and was superior (*p* < 0.05) to GTM. *Solanum myriacanthum* Dunal., *S. nudum* Dunal. and *S. seaforthianum* Andr. extracts demonstrated a similar effect (*p* > 0.05) to GTM. An increase of IFN-γ (*p* < 0.05) was displayed only by animals treated with *S. nudum* compared to the group treated with a vehicle, while no differences (*p* > 0.05) were observed for IL-12. **Conclusions:** *In vivo* effects of *Solanum* extracts were demonstrated, suggesting that this genus could be further explored as a new antileishmanial alternative.

## 1. Introduction

Leishmaniasis is a protozoal disease caused by more than 20 *Leishmania* species, belonging to Trypanosomatidae family. The parasite affects approximately 12 million people in 98 countries throughout the world [1,2]. The clinical manifestations are diverse and may range from ulcerative skin lesions and mucosal infections to visceral forms [3,4]. Presently, there is no vaccine available against leishmaniasis, and prophylactic measures for cutaneous leishmaniasis (CL) are ineffective [5,6].

Antimony compounds are a first-line drug treatment for leishmaniasis, including sodium stibogluconate and meglumine antimoniate. Drugs, such as miltefosine, liposomal amphotericin B, pentamidine and paromomycin, have also been used to treat this disease. However, severe adverse effects, high cost and drug resistance have hampered the treatment outcome of leishmaniasis [2,7]. During the last decades, there is a renewed interest worldwide in plants as pharmaceuticals [8], including search of new antileishmanial drugs [9,10].

In a previous study, Solanaceae plant extracts were evaluated for their antileishmanial activity. *Solanum* extracts displayed *in vitro* significant antileishmanial activities, particularly the ethanol extracts from the leaves of *S. havanense* Jacq. 1760, *S. myriacanthum* Dunal. 1813, *S. nudum* Dunal. 1816 and *S. seaforthianum* Andr. 1808 [11]. In this study, the *in vivo* efficacy of the extracts was studied in a BALB/c mice model infected with *L. amazonensis*. The effects on interferon gamma (IFN-γ) and interleukin 12 (IL-12) concentrations were also assessed. 

## 2. Materials and Methods

### 2.1. Parasite

*Leishmania amazonensis* MHOM/77BR/LTB0016 strain was kindly provided by the Department of Immunology from Oswaldo Cruz Foundation, Brazil. Parasites were maintained in lesions on footpads of BALB/c mice, routinely isolated and cultivated as promastigotes at 26 °C in Schneider’s Complete Medium, which consisted of Schneider medium (Sigma-Aldrich, St. Louis, MO, USA), 10% heat-inactivated fetal bovine serum (Sigma-Aldrich) and 100 μg of streptomycin/mL—100 U of penicillin/mL (Sigma-Aldrich). 

### 2.2. Plant Extracts

Samples of four species from *Solanum* genus identified by the taxonomist Victor Fuentes were used [11]. General characteristics, including voucher number, collection date and geographic area are listed in Table 1. The leaves of each plant were dried at 40 °C during 3 days, manually crushed (15–30 g) and afterwards, solid–liquid extractions were carried out by maceration at room temperature for 7 days with a mixture of EtOH:H_2_O (9:1, *v*:*v*). After 3, 5 and 7 days, supernatants were collected with a fresh solvent added on days 3 and 5, respectively. On day 7, the three individual extractions were combined (3, 5 and 7 days). The total extract was concentrated under a reduced pressure at 40°C in a rotoevaporator, subsequently lyophilized and dissolved in dimethylsulfoxide (DMSO) (BDH, Poole, England) at 20 mg/mL.

### 2.3. Reference Drugs

A pentavalent antimonial based on meglumine antimoniate, i.e., Glucantime^®^ (GTM) (Rhône-Poulenc Rorer, Mexico City, Mexico), was dissolved in sterile distilled water at 30 mg/mL and used as a reference drug. 

### 2.4. Animals

The Ethics Committee from the Institute of Tropical Medicine Pedro Kouri, Havana, Cuba, (Number 14–12) approved the protocol for the animal experiments. Healthy female BALB/c mice with a body weight between 20 and 22 g were obtained from the National Centre of Laboratory Animals Production (CENPALAB, Cuba). Animals were maintained according to “Guideline on the Care and Use of Laboratory Animals”. Ethical approval code: CEI-IPK 14-12, Date of approval: 25 September 2012.

### 2.5. Infection and Treatment

On Day 0, the BALB/c mice were infected with 50 μL of parasite suspension containing 5 × 10^6^ metacyclic promastigotes suspended in a sterile saline solution (SSS). The animals were injected subcutaneously in the right footpads. On day 30 post-infection (p.i.), the mice were randomly divided into seven different groups with eight animals in each group, which were weighted as a group. Then, the treatment was initiated with 50 µL of each *Solanum* extract or GTM at a final dose of 30 mg/kg dissolved in a mixture of SSS:DMSO (7:3, *v*:*v*). The products were injected intralesionally every 4 days at the infection site. A total of 5 doses were administrated to the BALB/c mice. In parallel, a control group (infected and untreated) and an infected and vehicle-treated group (SSS:DMSO (7:3, *v*:*v*)), using the same treatment regimen, were also included. 

### 2.6. Evaluation of Toxicity and Disease Progression after Treatment

The mice were observed daily, and death was recorded from the beginning of the treatment. The body weight of each group and the progression of the lesion size (by measuring infected and non-infected footpad swelling using a dial calliper) were weekly monitored from day 30 p.i. until day 74 p.i. (week 4 p.i. until week 10 p.i., respectively). On day 45 p.i. and day 74 p.i. (week 6 p.i. and week 10 p.i., respectively), three animals of each group were sacrificed using cervical dislocation, and the parasite burden in the infected area was determined. A sample of the lesion was excised and weighted, and a microtiter method was used [12]. Parasite burden was calculated as the logarithm of the positive dilution divided by the lesion weight.

### 2.7. Cytokine Measurement

In addition, on day 45 p.i., three samples of each animal group were obtained from the infected area. Tissues were homogenized in a lytic buffer, containing 1% of a Protease Inhibitor Cocktail (104 mM 4-(2-aminoethyl)benzenesulfonyl fluoride hydrochloride or AEBSF, 80 μM Aprotinin, 4 mM Bestatin, 1.4 mM E-64, 2 mM Leupeptin and 1.5 mM Pepstatin A), 1M Tris, 0.5 M EDTA, 1 M NaCl, 100 mM DTT and NP-40 (BD Biosciences, San Diego, CA, USA), and centrifuged at 14,000 rpm for 30 min. The supernatants of the samples were collected, and the presence of the cytokines IFN-γ (Cat. N°: 555138) and IL-12 (Cat. N°: 555165) were measured by an ELISA assay using pairs of monoclonal antibodies (BD OptEIA™; BD Biosciences, San Diego, CA, USA). The procedure was performed according to the manufacturer’s instructions.

### 2.8. Statistical Analyses

Data related to body weight, lesion size, parasite burden and cytokine concentration were collected in Microsoft Office Excel 2010 as a database, and afterwards, statistical analysis was performed. Moreover, the variation of body weight with respect to the start of the treatment was calculated. When the variation was higher than 10%, toxicity of the products should be considered. The data concerning the lesion progression and the parasite burden were analyzed with a variance test, followed by the Post Hoc Test (LDS test or planned comparison). Comparison of cytokine concentration was determined with the Mann–Whitney test. In all cases, *p*-values < 0.05 were considered statistically significant, and analyses were performed using a Statistical Windows Program (Release 4.5, StatSoft, Inc., New York, NY, USA, 1993). Finally, reduction of infection in percent related with disease control was calculated in each case, taking into account the lesion size and the parasite burden of the treated animals with extracts, a vehicle or GTM compared to the untreated control group on day 74 p.i. (week 10 p.i.). 

## 3. Results

In the animals treated with the *Solanum* extracts, neither death nor loss of body weight higher than 10% was observed. The follow-up of the body weight for the different groups is shown in Table 2.

**Figure 1:**
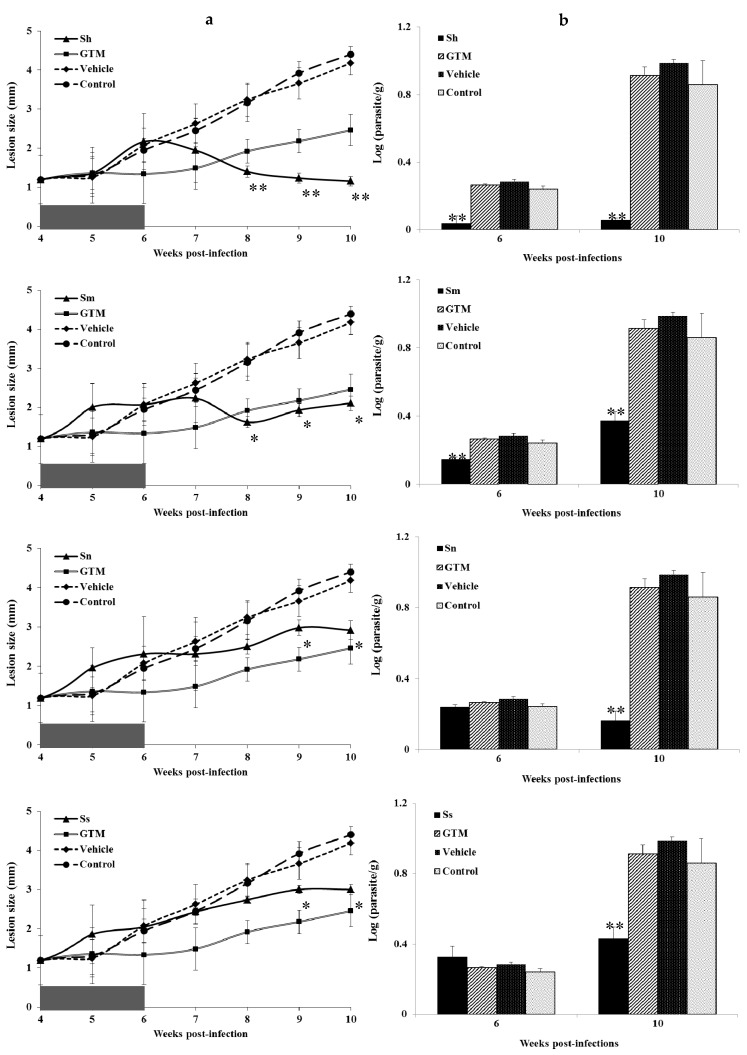
Effect of *Solanum* extracts on BALB/c mice infected subcutaneously with 5 × 10^6^ promastigotes of *L. amazonensis* in the footpad. The results were expressed as mean ± standard deviation. Treatment was started after 4 weeks p.i. Five doses were administered by intralesional route each 4 days. Glucantime^®^ (GTM) and extracts from *S. havanense* (Sh), *S. myriacanthum* (Sm), *S. nudum* (Sn) and *S. seaforthianum* (Ss) were administered at a dose of 30 mg/kg. (**a**) Lesion size; and (**b**) parasite burden. Vehicle: 50 µL of saline solution:dimethylsulfoxide (7:3, *v*:*v*); control: infected and untreated mice. * Statistically significant difference (*p* < 0.05) compared with the vehicle and the control group. ** Statistically significant difference (*p* < 0.05) compared with GTM, the vehicle and the control group.

Four weeks after the infection of the BALB/c mice with *L. amazonensis*, skin lesions at the inoculation site were visible, and the lesion size was similar (*p* > 0.05) in all the animals (Figure 1A). During the experiment, the two control groups showed a clear disease progression, and no significant lesion size (Figure 1A) differences (*p* > 0.05) were observed between the untreated mice and the mice treated with a vehicle. 

When the lesion size was analysed (Figure 1A), *S. havanense* was the most effective extract to resolve the parasite infection and was significantly better (*p <* 0.05) compared with GTM, and the vehicle-treated and the untreated groups. The animals treated with *S. myriacanthum* also demonstrated a positive effect, showing a smaller lesion size compared with GTM, although the lesion size difference did not differ significantly (*p* > 0.05). In contrast, the animals treated with *S. nudum* and *S. seaforthianum* extracts showed the biggest lesion size difference (*p <* 0.05) compared with GTM, although at the end of the experiment (week 9 p.i. and week 10 p.i.), significant differences (*p <* 0.05) were observed in comparison with the control animals.

**Figure 2:**
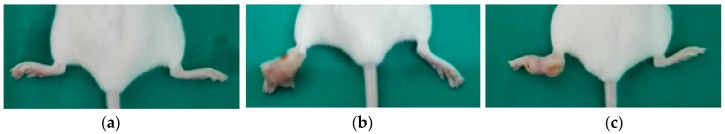
Pictures of footpads from BALB/c mice infected with *L. amazonensis* at 10 weeks p.i. (**a**) Animal treated with extract from *S. havanense*; (**b**) untreated animal; and (**c**) animal treated with GTM.

In addition to the lesion size, the parasite burden in the infected sites was also determined (Figure 1B). In this paper, all the cultured tissues were positive after microscopic examination, due to the presence of mobile promastigotes. Nevertheless, *S. havanense* was superior, showing a significantly lower parasite burden (*p* < 0.05), compared with GTM and the untreated animals at week 6 p.i. and week 10 p.i. This was also the case for the animals treated with *S. myriacanthum*. However, the mice treated with *S. nudum* and *S. seaforthianum* only showed a smaller parasite burden (*p* < 0.05) after 10 weeks p.i. compared with the other experimental groups. The efficacy of the treatment with *S. havanense* was clearly visible at the end of the experiment (Figure 2).

Finally, the reduction of infection in the treated groups compared to the untreated animals was summarized in Table 3, which displayed direct evidence on the resolution of infection according previous results at the end of experiment (10 weeks p.i.). The higher reduction of infection could be associated with the animals treated by *S. havanense.*

**Figure 3:**
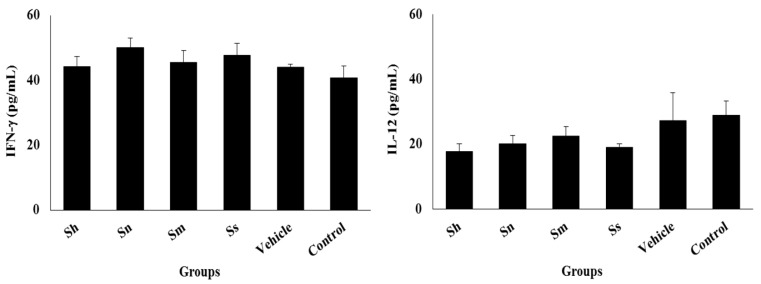
IFN-γ and IL-12 production in infected areas of BALB/c with *L. amazonensis*. The results were expressed as mean ± standard deviation. * Statistically significant difference (*p* < 0.05) compared with the vehicle-treated and the control groups.

To preliminarily evaluate the induction of Th1 response, the concentrations of IFN-γ and IL-12 were measured at week 6 p.i. in the lesion site of mice. In general, the treatments did not cause a significant effect on the cytokine concentrations compared with the control group (Figure 3). However, the animals treated with *S. nudum* showed statistically higher concentrations of IFN-γ (*p* < 0.05) compared to the vehicle-treated and the control groups. In the case of IL-12, slightly lower concentrations were observed for the extracts, but they did not differ (*p* > 0.05) from the vehicle-treated animals. 

## 4. Discussion

Due to the unsatisfactory current treatments of leishmaniasis, there is a strong need for new and effective antileishmanial drugs. In the search for innovative therapeutics, natural products are still an important source. *Solanum* genus is the most representative and largest genus from Solanaceae with more than 1700 species, which are mainly distributed in tropical regions. Many species belonging to this genus possess various pharmacological activities, including antibacterial [13], antifungal [14], antiviral [15] and antiparasitic activities [16,17]. In particular, antiprotozoal activity against the *Leishmania* parasite of the *Solanum* spp. has been previously reported [11,18,19,20]. In this study, we have evaluated four *Solanum* species for their activity against *L. amazonensis* in a BALB/c mouse infection model, which is known to be highly susceptible to *L. amazonensis* infection [21]. The extracts and the reference compound were administered through an intralesional injection, because it is more effective than an oral route [22]. In addition, a dose of 30 mg/kg was used, which is a dose close to the recommended dose for the reference drug, GTM, and previous positive results with other natural products [23,24].

In general, all the tested extracts were able to control the infection compared with the control and the vehicle-treated groups. *Solanum* genus was characterized by steroidal alkaloid saponins [25], although phytochemical studies of *Solanum* species have also reported a wide variety of different chemical entities, such as carotenoids, phenolic compounds [26], sesquiterpenoids [27] and coumarins [28]. All these different compounds displayed antileishmanial activity [10]. Moreover, havanine [29], acetyl ethiolin and solamarine [30] have been identified in *S. havanense*, while tumacones and tumacosides have been isolated from *S. nudum* [16].

Our animal experiments showed that only *S. havanense* extracts were superior to GTM; while *S. myriacanthum*, *S. nudum* and *S. seaforthianum* demonstrated a similar effect to GTM. A promising *in vitro* effect cannot always be correlated with a good *in vivo* effect, mainly due to the ADME properties of a compound. In the *in vitro* study, *S. nudum* and *S. seaforthianum* showed the lowest IC50-values against intracellular amastigotes (Table 1) [11], but when assessed *in vivo*, the group treated with these extracts exhibited a larger lesion size and a higher parasite burden compared with *S. havanense* and *S. myriacanthum*. The first possible explanation is related to a low absorption and a low distribution of compounds into subcutaneous tissues, since the compounds needed to be distributed to the tissues before interacting with the parasites. In this study, we selected intralesional administration because it is more probable that a major percent of active compounds arrive at parasite locations in comparison with oral, intraperitoneal or intravenous administration. However, compounds should be dissolved in subcutaneous tissues, be distributed among cells, enter into macrophage and other probably infect cells, such as dendritic cells, and finally perform the effect inside parasitophore vacuoles. Differences in the ability of compounds to dissolve, distribute and come across membrane are possible, and could be one reason of difference in activity observed. Secondly, the *in vivo* effects of the extracts could be associated with indirect actions, such as antioxidant, anti-inflammatory or immunostimulatory activities. To the best of our knowledge, previous pharmacological investigations of *S. havanense* have not been reported, while *Solanum lycopersicum* L. showed an antioxidant activity [31] and *Solanum melongena* L. possessed anti-inflammatory effects [32]. 

In summary, although a positive antileishmanial effect in the murine model was observed in the four tested extracts, activities were caused by different active principles. Currently, no identification of compounds has been carried out because the extracts are very complex mixtures of compounds. Previously, a metabolomics study using ultra-performance liquid chromatography with mass spectrometry (UPLC-MS) displayed variations in qualitative and semi-quantitative chemical compositions. In this sense, an extensive mass list in a chemical profile of extracts corresponding to a wide variety of metabolites was appreciated [11]. Then, in order to identify probable antileishmanial compounds, a bio-guide fractionation of extracts and standardization of respective chemical profile are needed. 

Considering that effective defense towards *Leishmania* strains, including *L. amazonensis*, depends strictly upon T (Th1) cells and macrophage activating cytokines [33,34], we measured the production of IFN-γ and IL-12. These cytokines initiated and/or drove the basic antileishmanial Th1 profile and played particularly prominent experimental roles. In the present study, we showed that only the *S. nudum* extract was able to slightly increase IFN-γ production, which is a key cytokine for protection against many infections caused by intracellular parasites [35,36,37]. The mechanism by which IFN-γ acts against intracellular parasites is through the classical activation of macrophages. Inducible nitric oxide synthase (iNOS) will be expressed, leading to the production of nitric oxide (NO) and consequently to the death of the parasites [38]. It has been previously postulated that *L. amazonensis* causes a strong subversion of the host immune response [39], whereby immunomodulating compounds could be used in disease control. The previously demonstrated direct antileishmanial activity of *S. nudum* [11] and the increase of IFN-γ can open further work to study the probable immunomodulator activity of the extract at different time after treatment and cytokines profile as a ratio (Th1/Th2). Probably, the delayed effect of *S. nudum* extracts that was observed during the last weeks of the in vivo experiments could be the result of its immunomodulatory effect. In addition, it is known that a protective immunity against all species of *Leishmania* is mediated by an IL-12-driven Th1 response to result in an increased IFN-g production [40]. Therefore, IL-12 is important for primary Th1 immunity against *Leishmania* to maintain a local Th1 response during infection [41,42]. However, in our study, lower IL-12 levels were observed in mice, including in the animals treated with *S. nudum*. Probably, the lower concentrations of IL-12 could be result of measurement time. 

Thus, we can suggest that the antileishmanial effect observed by *S. nudum* could be responsible for a direct parasite action and influenced by a probable immunomodulator activity, which should be demonstrated. Usually, the parasite load determined the level of cytokines and decreases with the infection, which was in correspondence with reduction of infection to animals treated with *S. nudum* that showed 80.8% of resolution of parasite burden. Nevertheless, an effective elimination of *L. amazonensis* parasites in BALB/c mice needed additional neutralization of both IL-10 and IL-4 cytokines in order for IFNγ to reach a threshold activity needed for parasite killing [43]. Similar results were discussed by Barroso and collaborators related to the immunomodulator effect of Z-100—a polysaccharide obtained from *Mycobacterium tuberculosis*—evaluated in the same model of CL in BALB/c mice infected with *L. amazonensis* [40].

In scientific literatures, some studies have divulged that leishmanicidal plants extracts can potentiate key cellular immune responses, probably due to plant secondary metabolites, which have proven to be coherent in the quest for more efficacious and less toxic antileishmanial drugs [44,45,46]. Then, plant-derived products have become a focal point for discovery of antileishmanials with leishmanicidal and immunomodulatory properties. In this sense, we cited per example: the extract from *Artemisia annua* L. [47], *Asparagus racemosus* Willd. [45] and *Kalanchoe pinnata* (Lam.) Pers. [48].

## 5. Conclusions

To conclude, our previous *in vitro* results of the *Solanum* extracts [11] and their *in vivo* effects showed that this genus could be considered as a potential antileishmanial alternative. Future determination of the chemical composition and standardization of the extracts are strongly suggested, which could be explored as a new alternative in the treatment of leishmaniasis, in particular, based on *S. havanense*-derived products. In addition, basic studies on the mechanisms of action and immunomodulatory activity were addressed.

## Figures and Tables

**Table 1:** General characteristics of plants from *Solanum* genus used in this study.

Plants Species	Locality ^1^	Collection Date	Voucher Specimen ^2^	In Vitro Activity ^3^
*S. havanense*	Guira de Melena, Artemisa	October, 2010	ROIG-4834	IC_50_ = 13.6 µg/mL CC_50_ = 268.4 µg/mL SI = 20
*S. myriacanthum*	Bahia Honda, Artemisa	July, 2010	ROIG-4821	IC50 = 11.2 µg/mL CC50 = 86.6 µg/mL SI = 8
*S. nudum*	Bahia Honda, Artemisa	July, 2010	ROIG-4822	IC_50_ < 6.25 µg/mL CC_50_ = 115.0 µg/mL SI > 18
*S. seaforthianum*	Santiago de las Vegas, La Habana	July, 2010	ROIG-4815	IC_50_ < 6.25 µg/mL CC_50_ = 194.3 µg/mL SI > 31

^1^ Municipality and province of Cuba where plants were collected; ^2^ Herbarium name: ROIG-Herbarium of Experimental Station of Medicinal Plants “Dr. Juan Tomás Roig”, Cuba; ^3^ Data from Monzote et al. 2016 [11]. IC_50_: concentration of drug that caused 50% of growth inhibition of *L. amazonensis* amastigotes. CC_50_: concentration of drug that caused 50% of mortality of peritoneal macrophage from BALB/c. SI: selectivity index (CC_50_ macrophage/IC_50_ amastigotes).

**Table 2:** Variation of body weight (%) of the animal groups infected with *L. amazonensis* and treated with *Solanum* extracts, a vehicle or a reference drug.

Groups	Variation of Body Weight (%) ^1^					
	5 w.p.i.	6 w.p.i.	7 w.p.i.	8 w.p.i.	9 w.p.i.	10 w.p.i.
*S. havanense*	0.1	2.6	−2.7	−0.6	2.8	6.1
*S. myriacanthum*	−0.1	0.0	−2.0	−1.9	−1.6	−0.8
*S. nudum*	1.0	−0.2	−2.0	−1.9	−0.3	0.8
*S. seaforthianum*	−1.2	−2.6	−4.6	−4.4	−4.0	−1.5
GTM ^2^	−2.3	−2.9	−3.9	−4.7	−3.9	−4.5
Vehicle ^3^	−1.6	−2.4	−4.4	−3.1	−4.1	−1.3
Control ^4^	0.3	6.9	2.5	0.3	2.8	1.8

w.p.i.: weeks post-infection. ^1^ Positive number represents increase of body weight and negative number means decrease of body weight with respect to week 4 p.i. (Start of the treatment); ^2^ GTM: Glucantime^®^—reference drug; ^3^ Vehicle: Saline solution:dimethylsulfoxide (7:3, *v*:*v*). ^4^ Control: Infected and untreated mice.

**Table 3:** Reduction of infection (%) of the animal groups infected with *L. amazonensis* and treated with *Solanum* extracts, a vehicle or a reference drug.

Groups	Reduction of Infection (%)	
	Lesion Size	Parasite Burden
*S. havanense*	73.7	93.6
*S. myriacanthum*	52.0	56.8
*S. nudum*	33.6	80.8
*S. seaforthianum*	31.8	49.9
GTM ^1^	44.1	0
Vehicle ^2^	5.0	0

^1^ GTM: Glucantime^®^—reference drug; ^2^ Vehicle: Saline solution:dimethylsulfoxide (7:3, *v*:*v*).
